# Profound Presentation of Retinopathy in a Patient with Sickle Cell Trait and Diabetes Mellitus

**DOI:** 10.18502/jovr.v15i1.5962

**Published:** 2020-02-02

**Authors:** Gautam Vangipuarm, Steven S. Saraf, Qinqin Zhang, Ruikang Wang, Kasra A Rezaei

**Affiliations:** ^1^Department of Ophthalmology, University of Washington, Seattle, WA, USA; ^2^Department of Bioengineering, University of Washington, Seattle, WA, USA

##  PRESENTATION 

**Figure 1:**
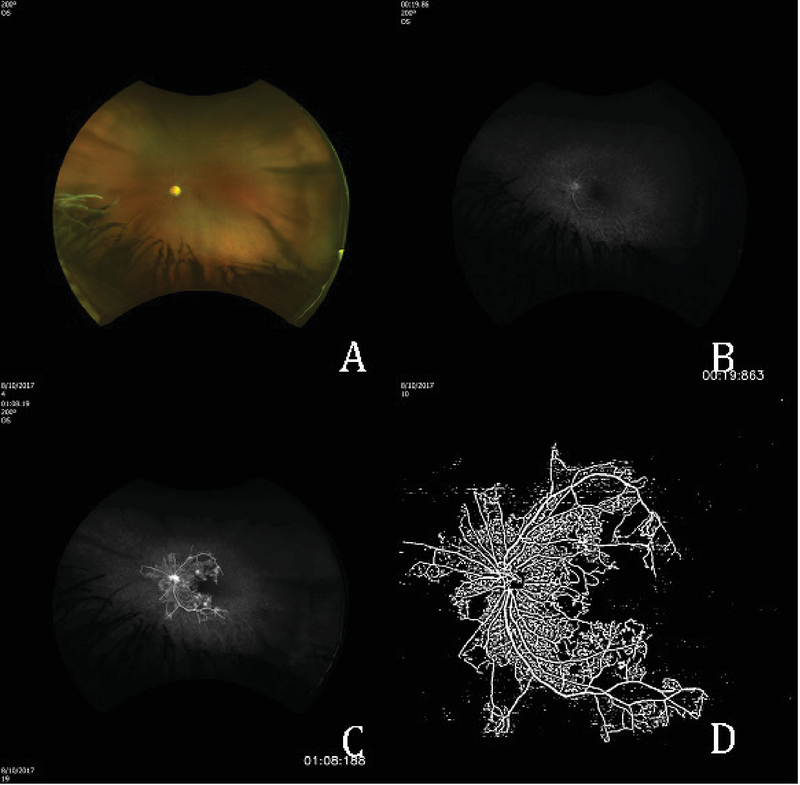
Color fundus photo, left eye (A) early (B) and late (C) fluorescein angiography of the left eye showing marked peripheral ischemia and posterior pole neovascularization. OCT angiography (D) showing severely decreased vascular density.

A 43-year-old functionally monocular African American woman with longstanding type 2 diabetes mellitus presented for care of her better-seeing left eye. Originally suspected of having proliferative diabetic retinopathy (PDR) as the cause of her bilateral visual impairment, fluorescein angiography and optical coherence tomography angiography revealed a marked peripheral non-perfusion which was out of proportion for a typical diabetic retinopathy (Figure 1). A comprehensive uveitic and vasculopathic workup was therefore initiated. The workup was largely negative except for hemoglobin electrophoresis, which was consistent with the sickle cell trait (or hemoglobinopathy) (Table 1). The patient was counseled on her diagnosis and continues to be treated with laser photocoagulation for her peripheral neovascularization.

**Table 1:** Laboratory assessment of other etiologies for extensive peripheral non-perfusion including pro-thrombotic and vasculitic causes


**Test ordered**	**Result (normal range)**
Angiotensin converting enzyme (U/L)	26U/L (8–53 U/L)
Anti-nuclear antibody	Negative
Cryoglobulin	Negative
Erythrocyte sedimentation rate (mm/H)	60 mm/H **high** (0–20 mm/H)
HIV Ag and Ab	Nonreactive
Anti-myeloperoxidase	Negative
Anti PR3	Negative
Rheumatoid factor	< 10
Serologic syphilis panel	Negative
Anti-thrombin activity	123% (normal)
C-reactive protein (mg/L)	24.9 mg/L **high **(0–10 mg/L)
Activated protein S (%)	113% (65–150%)
Activated protein C (%)	121% (55–150%)
Factor V Leiden	Negative
Homocysteine	Negative
Prothrombin time (s)	14.1 s (10.7–15.6 s)
INR (s)	1.1 s (0.8–1.3 s)
CBC	Normal
CMP	Glucose 353 mg/dL (62–125 mg/dL)
Herpes type 1&2 serology	Positive for HSV-1 and HSV-2
CMV (serum antibody)	Positive
Hemoglobin electrophoresis	Consistent with HbS trait
Quantiferon-TB Gold	Negative
	
	
CBC, complete blood count; CMP, comprehensive metabolic panel; CMV, antibodies to cytomegalovirus; HIV, human immunodeficiency virus; INR, international normalized ratio; mg/dL, milligrams per deciliter; mm/H, millimeter per hour U/L, Units Per Liter	

##  DISCUSSION 

This report strengthens the hypothesis that diabetic retinopathy and coexisting vasculopathic diseases, even sickle cell trait, may have a synergistic effect on the overall disease burden. A broad differential must be maintained in patients with presumed diabetic retinopathy, especially those with uncharacteristic imaging findings.^[1,2,3,4,5]^

##  Financial Support and Sponsorship

This study was supported by the Department of Ophthalmology, University of Washington.

##  Conflicts of Interest

There are no conflicts of interest.
